# Provider confidence in the telemedicine spine evaluation: results from a global study

**DOI:** 10.1007/s00586-020-06653-8

**Published:** 2020-11-22

**Authors:** Francis Lovecchio, Grant J. Riew, Dino Samartzis, Philip K. Louie, Niccole Germscheid, Howard S. An, Jason Pui Yin Cheung, Norman Chutkan, Gary Michael Mallow, Marko H. Neva, Frank M. Phillips, Daniel M. Sciubba, Mohammad El-Sharkawi, Marcelo Valacco, Michael H. McCarthy, Melvin C. Makhni, Sravisht Iyer

**Affiliations:** 1 Department of Orthopaedic Surgery, Hospital for Special Surgery, New York, NY USA; 2 Department of Orthopaedic Surgery, Brigham and Women’s Hospital, Harvard Medical School, Boston, MA USA; 3 Department of Orthopaedic Surgery, Rush University Medical Center, Chicago, IL USA; 4 The International Spine Research and Innovation Initiative, Rush University Medical Center, Chicago, IL USA; 5 Neuroscience Institute, Virginia Mason Medical Center, Seattle, WA USA; 6 Research Department, AO Spine International, Davos, Switzerland; 7 Department of Orthopaedics & Traumatology, The University of Hong Kong, Hong Kong SAR, China; 8 Department of Orthopaedic Surgery, University of Arizona College of Medicine, Phoenix, AZ USA; 9 Department of Orthopaedic and Trauma Surgery, Tampere University Hospital, Tampere, Finland; 10 Department of Neurosurgery, Baltimore, MD, USA, John Hopkins University, Baltimore, MD USA; 11 Department of Orthopaedic and Trauma Surgery, Assiut University Medical School, Assiut, Egypt; 12 Department of Orthopaedics, Churruca Hospital de Buenos Aires, Buenos Aires, Argentina; 13 Indiana Spine Group, Carmel, IN USA

**Keywords:** Telemedicine, Spine surgery, Examination, International, Survey

## Abstract

**Purpose:**

To utilize data from a global spine surgeon survey to elucidate (1) overall confidence in the telemedicine evaluation and (2) determinants of provider confidence.

**Methods:**

Members of AO Spine International were sent a survey encompassing participant’s experience with, perception of, and comparison of telemedicine to in-person visits. The survey was designed through a Delphi approach, with four rounds of question review by the multi-disciplinary authors. Data were stratified by provider age, experience, telemedicine platform, trust in telemedicine, and specialty.

**Results:**

Four hundred and eighty-five surgeons participated in the survey. The global effort included respondents from Africa (19.9%), Asia Pacific (19.7%), Europe (24.3%), North America (9.4%), and South America (26.6%). Providers felt that physical exam-based tasks (e.g., provocative testing, assessing neurologic deficits/myelopathy, etc.) were inferior to in-person exams, while communication-based aspects (e.g., history taking, imaging review, etc.) were equivalent. Participants who performed greater than 50 visits were more likely to believe telemedicine was at least equivalent to in-person visits in the ability to make an accurate diagnosis (OR 2.37, 95% C.I. 1.03–5.43). Compared to in-person encounters, video (versus phone only) visits were associated with increased confidence in the ability of telemedicine to formulate and communicate a treatment plan (OR 3.88, 95% C.I. 1.71–8.84).

**Conclusion:**

Spine surgeons are confident in the ability of telemedicine to communicate with patients, but are concerned about its capacity to accurately make physical exam-based diagnoses. Future research should concentrate on standardizing the remote examination and the development of appropriate use criteria in order to increase provider confidence in telemedicine technology.

## Introduction

Though COVID-19 accelerated the worldwide adoption of remote health care, recent trends had already established telemedicine as a fast-growing clinical tool [1]. The last two decades have witnessed the expansion of telemedicine in general surgery, medical education, and surgical subspecialties [2, 3], a trend now cemented in place by current COVID-19 restrictions. Spine surgery has also experienced similar growth and adoption of this practice change [4].

Certain challenges are inevitable in the widespread adoption of any “new” mode of care delivery, especially with the incorporation of new technology. Barriers to the adoption of telemedicine in spine surgery include the implementation of novel technology, lack of technological literacy, new medicolegal territory, negative financial implications, and regulatory concerns [5]. However, as with any new technology, the largest barrier to widespread adoption is user confidence in the clinical utility of telemedicine. Whether telemedicine will play a large or small part in the overall patient evaluation remains to be seen. One controversial aspect of telemedicine is the ability to perform accurate physical examination, as experts diverge on how best to examine patients and how much faith can be placed in these virtual evaluations [6–8].

Understanding surgeon confidence in telemedicine will be necessary to develop appropriate use criteria, improve deficient areas, and standardize the telemedicine evaluation. International perspectives are particularly valuable as they offer the widest array of experience and opinion. The purpose of this study was to utilize data from a global spine surgeon survey to elucidate (1) overall confidence in the telemedicine evaluation and (2) determinants of provider confidence in this novel method of patient evaluation.

## Methods

### Survey design

The data utilized in this study were retrieved from a cross-sectional, global survey designed to capture spine surgeons’ perspectives on telemedicine. The survey was designed through a Delphi approach, in which all questions underwent four rounds of review by the multi-disciplinary study authors [9]. The final survey consisted of 42 questions encompassing seven major categories: demographics, usage of telemedicine, patient perception, trust in telemedicine, challenges and benefits, comparison to in-person visits, and training and research (Appendix 1). The survey was anonymous, as no identifying information was collected from respondents.

### Study sample

The survey entitled “Telemedicine & the Spine Surgeon—Perspectives and Practices Worldwide” was distributed through email to members of AO Spine starting May 15, 2020, with closure of the survey on May 31, 2020. AO Spine is the largest international society dedicated to spine surgery, consisting of over 30,000 professionals, of which greater than 6000 are practicing spine surgeons (www.aospine.org). The survey was distributed to the 3805 surgeons who opted in to receive email surveys.

### Statistical analyses

Survey respondents were grouped into five geographical regions: Africa, Asia–Pacific, Europe, North America, and South America. All questions were considered optional; thus, pairwise deletion was utilized for missing data points. In order to present the large content of the survey in a concise manner, statistical analyses were divided into four major themes: global perspectives, challenges and benefits, telemedicine evaluation, and training and research. Thus, the current analysis focuses specifically on survey questions pertaining to the telemedicine evaluation.

The data were stratified by region, provider age, telemedicine platform, experience with telemedicine, specialty, and trust in telemedicine, and compared. To determine participants’ sense of trust in telemedicine, the survey query: “If you or a family member were a patient, do you believe the initial visit can be performed through telemedicine?" was utilized as an anchor question, with a response of “agree” or “strongly agree” classified as a positive response. Likert scale questions were analyzed as both categorical and continuous variables. Categorical variables were compared using Pearson’s Chi-square test and continuous variables were compared using Mann–Whitney U tests or ANOVA test as appropriate. To control for confounding and isolate factors independently associated with confidence in the telemedicine evaluation, multivariate logistic regressions were performed for each survey question. Odds ratios (OR) and 95% confidence intervals (CI) were assessed for precision. Hosmer–Lemeshow tests were used to ensure appropriate goodness of fit for all models. All statistical analyses were performed using SPSS version 25.0 (IBM, Armonk, NY), and the threshold for statistical significance was established at *p* < 0.05.

## Results

### Survey sample

In total, 485 spine surgeons participated in the survey. Most survey participants were between 35–44 (68.7%) and 45–54 (33.0%) years old, and the vast majority were male (94.5%). Responses were split between Africa (19.9%), Asia Pacific (19.7%), Europe (24.3%), North America (9.4%), and South America (26.6%). Videoconferencing platforms (EMR-integrated or non-secure) were utilized by 57.5%, while 34.6% of respondents used telephone calls only for telemedicine. At the time of the survey, most participants had performed fewer than 50 visits (77.8%), with less than a quarter of having performed over 50 telehealth visits (22.2%) (Table 1).

**Table 1:** Survey respondent demographics

	*n*	Percent^a^		*n*	Percent
Sex			Specialty		
Male	446	94.5%	Orthopedics	332	68.5%
Female	26	5.5%	Neurosurgery	144	29.7%
Age (years)			Trauma	50	10.3%
25–34	56	11.7%	Pediatric surgery	16	3.3%
35–44	173	36.1%	Other	14	2.9%
45–54	160	33.4%	Years practicing spine surgery		
55–64	73	15.2%	0–5	100	20.9%
65 +	17	3.5%	5–10	116	24.3%
Geographic region			11–15	82	17.2%
Africa	95	19.9%	16–20	68	14.2%
Asia Pacific	94	19.7%	20 +	112	23.4%
Europe	116	24.3%	Telemedicine platform		
North America	45	9.4%	Phone	100	34.6%
South America	127	26.6%	Video	116	57.5%
Estimated population your hospital serves			Total number of telemedicine visits performed		
< 100,000	46	9.6%	0–10	57	24.2%
100,000–500,000	118	24.7%	11–25	75	31.8%
500,000–1,000,000	100	21.0%	25–50	52	22.0%
1,000,000–2,000,000	67	14.0%	51–100	27	11.4%
> 2,000,000	146	30.6%	100 +	25	10.6%
Hospital community			Trust in telemedicine anchor question^b^		
Urban	408	85.4%	Agree	356	74.9%
Suburban	63	13.2%	Neutral or disagree	100	21.1%
Rural	7	1.5%			
Practice type					
Academic/university hospital	164	34.0%			
"Privademic" (academic/private combined)	128	26.6%			
Private group, < 10 practitioners	58	12.0%			
Private group, > 10 practitioners	20	4.1%			
Individual practice	35	7.3%			
Government/military hospital	34	7.1%			
Hospital employee	29	6.0%			
Other	14	2.9%			

^a^Percentages calculated based on number of responses to question, not overall survey

^b^Anchor question: "If you or a family member were a patient, do you believe the initial visit can be performed through telemedicine?"

**Table 2:** Equivalency of telemedicine evaluation and entire sample

	*N* respondents	Much worse	Slightly worse	Equivalent	Slightly better	Much better
How does telemedicine compare to in-person visits for the ability to…						
Take a patient history	221	4.5%	31.2%	56.1%	4.1%	4.1%
Localize pain	219	19.2%	54.3%	24.7%	1.4%	0.5%
Assess neurologic deficits	221	57.9%	36.7%	2.3%	1.8%	1.4%
Assess myelopathy	218	52.3%	36.7%	9.2%	0.9%	0.9%
Assess spinal deformity	220	27.3%	45.5%	24.5%	1.4%	1.4%
Perform provocative tests (straight leg raise, Spurling's, Lhermitte's)	220	50.9%	38.6%	8.6%	0.9%	0.9%
Review imaging and explain patients	220	5.0%	22.7%	54.1%	13.6%	4.5%
Make an accurate diagnosis	220	15.9%	54.1%	26.8%	2.7%	0.5%
Formulate and communicate a treatment plan	220	3.6%	28.6%	57.7%	8.2%	1.8%

**Table 3:** Confidene in telemedicine evaluation, stratified by age, platform, experience and speciality

	Overall respondents		Age > 55 (*n* = 36)		Age > 55 (*n* = 184)		*p* Value	Phone (*n* = 73)		Video (*n* = 133)		*p* Value	Visits ≤ 50(*n* = 171)		Visits < 50(*n* = 49)		*p* Value	Ortho (*n* = 154)		Neuro (*n* = 60)		*p* Value
	Mean (SD^a^)		Mean (SD^a^)		Mean (SD^a^)			Mean (SD^a^)		Mean (SD^a^)			Mean (SD^a^)		Mean (SD^a^)			Mean (SD^a^)		Mean (SD^a^)		
How does telemedicine compare to in-person visits for the ability to…																						
Take a patient history	−0.28	0.79	−0.28	0.74	−0.28	0.80	0.996	−0.37	0.81	−0.23	0.77	0.152	−0.29	0.81	−0.24	0.72	0.890	−0.27	0.81	−0.30	0.67	0.835
Localize pain	−0.90	0.73	−0.94	0.67	−0.89	0.74	0.779	−1.01	0.70	−0.85	0.75	0.127	−0.93	0.72	−0.84	0.75	0.374	−0.87	0.73	−0.93	0.72	0.603
Assess neurologic deficits	−1.48	0.75	−1.58	0.55	−1.46	0.79	0.565	−1.58	0.62	−1.42	0.83	0.239	−1.49	0.78	−1.45	0.68	0.429	−1.52	0.69	−1.37	0.92	0.501
Assess myelopathy	−1.39	0.77	−1.39	0.64	−1.38	0.79	0.718	−1.42	0.78	−1.37	0.77	0.527	−1.39	0.75	−1.36	0.85	0.971	−1.40	0.76	−1.34	0.76	0.543
Assess spinal deformity	−0.96	0.83	−0.81	0.75	−0.98	0.85	0.120	−1.05	0.86	−0.89	0.83	0.165	−0.96	0.83	−0.96	0.87	0.977	−0.95	0.85	−0.97	0.76	0.909
Perform provocative tests (straight leg raise, Spurling's, Lhermitte's)	−1.38	0.76	−1.39	0.69	−1.37	0.77	0.929	−1.49	0.75	−1.32	0.77	0.070	−1.38	0.74	−1.39	0.84	0.682	−1.35	0.75	−1.45	0.72	0.300
Review imaging and explain patients	−0.10	0.86	−0.00	0.89	−0.12	0.86	0.738	−0.27	0.89	−0.02	0.86	0.024	−0.13	0.83	−0.02	0.97	0.709	−0.20	0.90	−0.10	0.71	0.019
Make an accurate diagnosis	−0.82	0.74	−0.92	0.55	−0.80	0.77	0.409	−0.92	0.74	−0.74	0.74	0.112	−0.88	0.69	−0.63	0.88	0.049	−0.88	0.70	−0.65	0.80	0.080
Formulate and communicate a treatment plan	−0.24	0.73	−0.25	0.77	−0.24	0.72	0.693	−0.41	0.68	−0.08	0.69	<0.001	−0.32	0.69	−0.02	0.80	0.018	−0.27	0.73	−0.20	0.73	0.320

^a^Standard deviation

**Table 4:** Confidence in telemedicine evaluation, stratified by provider trust in telemedicine

	Initial visit can be done through telemedicine (*n* = 123)		No initial visit through telemedicine (*n* = 96)		*p* value
	Mean (SD^a^)		Mean (SD^a^)		
How does telemedicine compare to in-person visits for the ability to… (-2 Telemedicine much worse, -1 slightly worse, 0 equivalent, 1 slightly better, 2 much better)					
Take a patient history	−0.12	0.81	−0.49	0.73	0.002
Localize pain	−0.80	0.72	−1.04	0.80	0.010
Assess neurologic deficits	−1.41	0.71	−1.57	2.3%	0.004
Assess myelopathy	−1.33	0.79	−1.46	0.74	0.144
Assess spinal deformity	−0.76	0.83	−1.20	0.78	< 0.001
Perform provocative tests	−1.26	0.81	−1.53	0.66	0.007
Review imaging and explain to patients	−0.01	0.89	−0.20	0.82	0.090
Make an accurate diagnosis	−0.67	0.67	−1.02	0.78	< 0.001
Formulate and communicate a treatment plan	−0.10	0.70	−0.42	0.74	0.003

^a^ Standard deviation

**Table 5:** Independent predictors of confidence in telemedicine evaluation

Outcome of interest for multivariate model	Significant covariates	Odds ratio	95% Confidence interval	*p* value	Hosmer–Lemeshow
Compared to in-person visits, telemedicine is equivalent or better to…					
Take a patient history	None				0.85
Localize pain	None				0.38
Assess neurologic deficits*	.–				
Assess myelopathy	None				0.13
Assess spinal deformity	Age > 55	2.51	1.02–6.15	0.045	0.85
Perform provocative tests	None				0.74
Review imaging and explain patients	Neurosurgery	3.67	1.39–9.71	0.009	0.59
Make an accurate diagnosis	Visits > 50	2.37	1.03–5.43	0.042	0.32
Formulate and communicate a treatment plan	Video	3.88	1.71–8.84	0.001	0.91

Multivariate models controlled for age (> 55 vs. ≤ 55), platform (video vs. phone), provider experience (> 50 vs. ≤ 50 visits), region, specialty (orthopedic vs. neurosurgery, trauma/peds/other excluded), type of practice (academic/military/hospital employee vs. private/privademic), size of hospital's community, and type of hospital community. *Multivariate model not assessed given < 10 events per variable

### Confidence in the telemedicine evaluation—overall responses

**Fig. 1:**
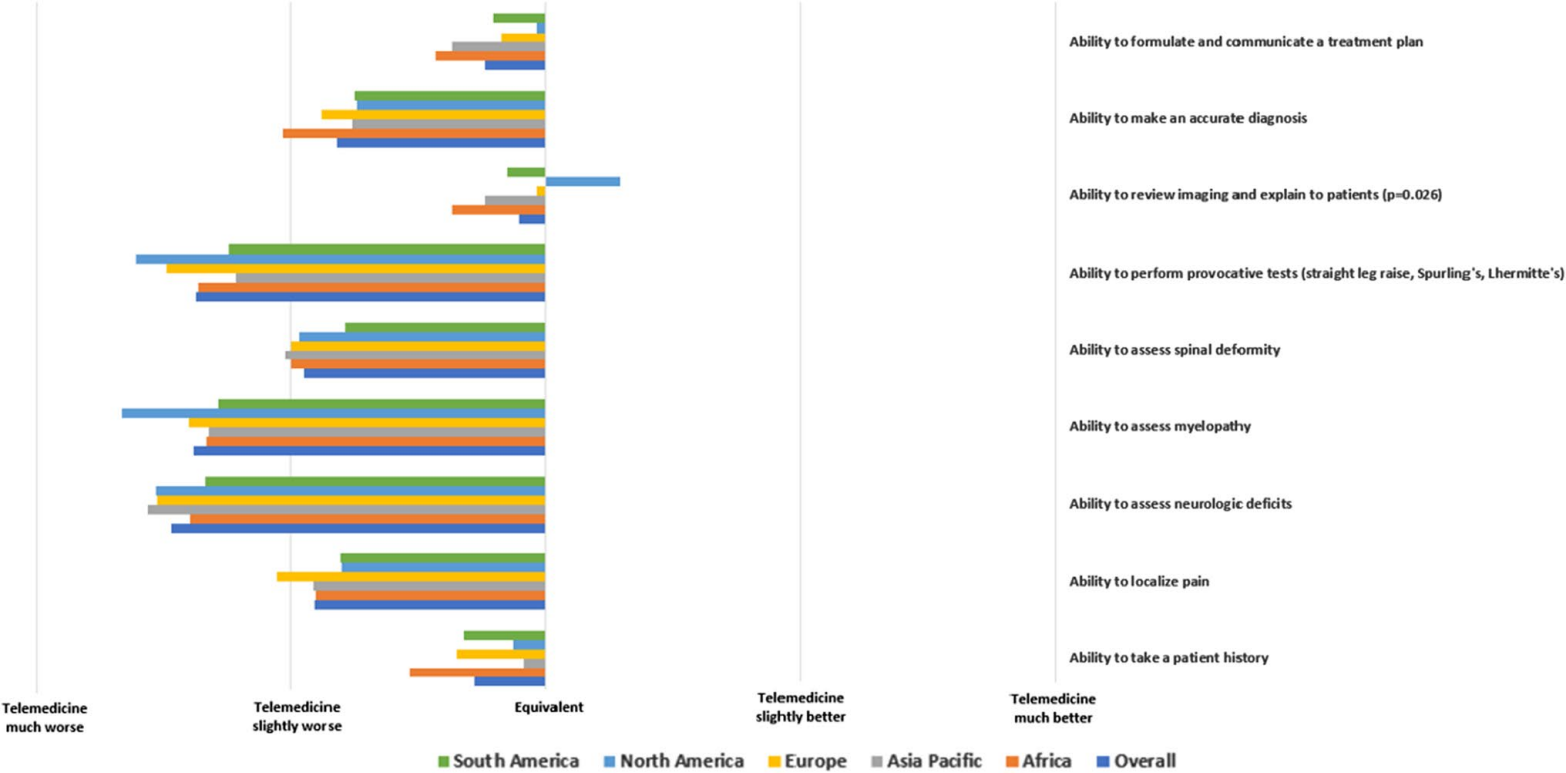
Provider confidence in various patient evaluation tasks, stratified by region. There were no significant differences in responses by region (*p* > 0.05 for all comparisons)

**Fig. 2:**
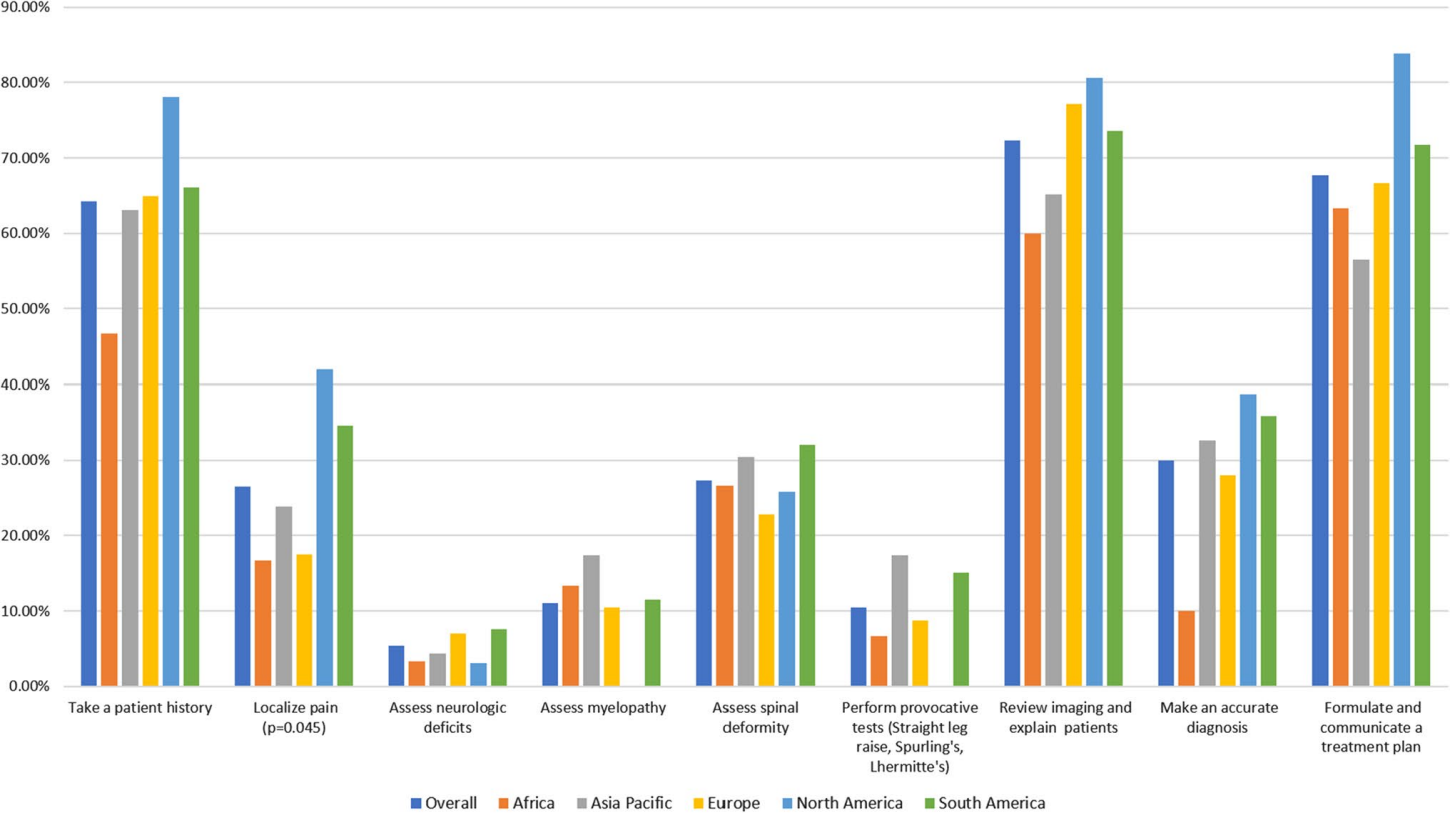
Equivalency of telemedicine for patient evaluations, stratified by region. The only significant difference was noted in the equivalency of telemedicine to localize pain (*p* = 0.045, *p* > 0.05 for all other comparisons)

Participants were asked to compare telemedicine to in-person visits among multiple facets of the patient evaluation (i.e., history taking, examination, imaging review, diagnosis). Analysis of the mean Likert scale responses demonstrated an overall leftward skew of provider opinion toward telemedicine evaluation (Fig. 1, Table 2). Most respondents felt that telemedicine was at least equivalent to in-person visits for communicative tasks, such as taking a patient history (64.3%), reviewing and explaining imaging (72.3%), and formulating and communicating a treatment plan (67.7%). Respondents had significantly less confidence in the physical exam portions of the evaluation; telemedicine was worse or much worse in assessing neurologic deficits (94.6%), provocative testing (89.5%) and myelopathy (89.0%) (Fig. 2 and Table 2).

### Region

In almost all analyses, region was not associated with differences in participant confidence in telemedicine (Figs. 1, 2). The significant differences noted in univariate analysis were all small and not significant after multivariate adjustment.

### Age

On univariate analyses, participant age was not found to be associated with confidence in the telemedicine evaluation (Table 3). However, multivariate adjustment demonstrated that participants > 55 years old were more likely to believe telemedicine was equivalent or better to in-person visits for the assessment of spinal deformity (OR: 2.51, 95% CI: 1.02–6.15) (Table 5).

### Telemedicine Platform

Compared with telephone (audio only), the use of videoconferencing technology was associated with increased confidence in the ability of telemedicine to formulate and communicate a treatment plan when compared to in-person visits (−0.08 ± 0.69 vs. −0.41 ± 0.68, *p* < 0.001) (Table 3). This relationship was sustained in the multivariate model (OR: 3.88, 95% CI: 1.71–8.84) (Table 5).

### Provider experience

Providers who had performed > 50 telemedicine visits demonstrated increased confidence in the ability of telemedicine to formulate and communicate a treatment plan (0.02 ± 0.80 vs. −0.32 ± 0.69, *p* = 0.018) and make an accurate diagnosis (−0.63 ± 0.88 vs. −0.88 ± 0.69, *p* = 0.049) (Table 3). On multivariate analysis, respondents experienced with telemedicine were more likely to believe telemedicine was equivalent or better than in-person visits in the ability to make an accurate diagnosis (OR: 2.37, 95% CI: 1.03–5.43).

### Specialty

Univariate analyses did not support a difference between orthopedic and neurosurgery in participant confidence in telemedicine evaluation. However, those with neurosurgery training described increased confidence in telemedicine for imaging review and explanation (OR: 3.67, 95% CI: 1.39–9.71), compared to their orthopedic-trained colleagues.

### Provider trust in telemedicine

Our anchor question for provider trust in telemedicine revealed that most respondents (74.9%) believed that the initial visit could be performed through telemedicine. An affirmative response to the anchor question was associated with slightly increased confidence in telemedicine in almost all facets of the patient evaluation, with the exception of the assessment of myelopathy and imaging review (Table 4). However, none of these relationships were sustained in the multivariate analyses (Table 5).

## Discussion

Our global survey of 485 spine surgeons demonstrates that the use of telemedicine in spine surgery is still in the early stages, with most participants having conducted relatively few telehealth visits at the time of the survey (Table 1). Overall, there remains considerable skepticism. Specifically, compared to in-person visits, spine surgeons generally felt that telemedicine was inferior for every aspect of the patient evaluation (Fig. 1). However, there was a clear divergence between communication- and examination-based aspects of the patient evaluation.

The majority of survey respondents felt that telemedicine was at least equivalent to in-person visits for taking a patient history, reviewing and explaining imaging, and formulating and communicating a treatment plan (Fig. 2, Table 2). Although telemedicine remains in the early stages of adoption, these data are largely in keeping with the existing literature [10–13]. Agha et al. [12] conducted a non-inferiority, randomized controlled trial (RCT) of 221 patients seen in a medical office. They used a validated questionnaire to demonstrate that patient-centered communication tasks and clinical competence were perceived equally regardless of visit type (in-person or telemedicine). On the physician side, Buvik et al. [13] surveyed orthopedic surgeons and found that provider assessment of communication was nearly identical between remote and in-person orthopedic consultations.

Importantly, both of these studies used videoconferencing. Additionally, because these studies were conducted by early adopters of telemedicine, it is likely that these providers were more experienced with telemedicine. These factors (the use of video and prior experience) were independently associated with increased confidence in telemedicine in our survey (Table 4).

Taken together, these data suggest that (when feasible) video conferencing should be the recommended platform for telemedicine visits. Additionally, as institutions integrate telemedicine into their practices, it will be important to provide exposure to this new technology in a graduated fashion. When possible, institutional adoption of telemedicine should include training on the differences between telemedicine and in-person visits, simulated visits, exposure of house staff to telemedicine and accounting for an “adjustment phase” for new providers [6, 7, 16], similar to the steps taken when onboarding a new provider to the practice.

In contrast to communication-based tasks, surgeon confidence in physical-exam-based patient evaluation (e.g., assessment of myelopathy, neurologic deficits, and provocative testing) was universally low. Almost all physicians (~ 90%) considered telemedicine slightly to be much worse than in-person visits for these tasks.

The inability of telemedicine to replicate the hands-on physical exam is probably the largest limitation to its adoption [8]. Interestingly, the need for “advanced physical exam maneuvers” has even been employed as an exclusion criterion from participation in orthopedic telemedicine RCTs [13]. Several authors have suggested strategies to improve the remote physical exam: asking patients to use weights (or body-weight maneuvers) and self-rate the required effort [6, 14], using composite movements [15] and utilizing technology to make reproducible measurements [16].

While our survey results leave little doubt about the telemedicine physical exam, it is certainly possible that a good patient history, imaging review, and a limited physical exam might be sufficient to make an accurate initial diagnosis in some patients [17]. In the situation where the diagnosis is not clear or surgical intervention is warranted, the provider may consider a follow-up in-person evaluation to supplement the telemedicine encounter. Certainly, there appears to be a learning curve to this type of visit (Table 5). In this sense, we expect providers to become more facile with their own telemedicine-based indications. Just as with any “new” technology, appropriate use criteria will have to be created for telemedicine, and the COVID19 pandemic makes this task ever more urgent. Expert consensus and high-quality research in this area are essential.

Our analysis is not without limitations. While there were several strengths of our survey population (international cohort split between regions, wide age range and years of practice, multiple spine surgery specialties and levels of experience with telemedicine, etc.), we only made the survey available for two weeks, capturing only a small portion of AO Spine members as a whole (485 [12.7%] of 3805) and all response items were optional. Thus, the population may have strong opinions about telemedicine which influenced their decision to participate. In addition, the participants were mainly from large, urban areas; these are populous regions that have traditionally not relied on telemedicine [18]. Furthermore, the timing of the survey (during COVID-19) must be considered when interpreting our findings. It is difficult to predict whether surgeon opinion on telemedicine will change once the pandemic is under better control, especially if pre-pandemic regulations are re-instituted [5]. Finally, the study was not powered to rule out associations; thus, negative findings should be interpreted cautiously.

## Conclusions

In conclusion, our global survey demonstrated that spine surgeons were confident in the ability of telemedicine to communicate with patients, but were concerned about its capacity to accurately make physical exam-based diagnoses. Video technology was strongly associated with increased confidence and should be used when available. Provider experience allows for increased confidence in how and when to use telemedicine. Given the limitations in the telemedicine physical exam, further research is needed to standardize physical examination methods and develop appropriate use criteria for this new tool.

## Appendix 1: Survey

### Telemedicine & the Spine Surgeon – Spine Surgeon Perspectives and Practices Worldwide

**Purpose:** Given the need for social distancing with COVID-19, telemedicine services have expanded around the world. We define telemedicine in this survey as clinical care provided remotely through videoconferencing or telephone. This survey seeks to determine:

1. 1.The extent of spine surgeon adoption of telemedicine
2. Satisfaction with telemedicine
3. Comparison of telemedicine to in-person visits
4. The use of telemedicine in research and training
5. Variations in perspectives and practices worldwide

The survey will not take more than 5–10 min to complete.Information obtained from this survey will be kept strictly confidential. The identity of all survey participants will remain anonymous. The findings of this survey will be disseminated via social media, journals, and other media platforms. Deadline to respond is May 31, 2020. Thank you in advance for taking the time to fill out this survey.
